# Linkage between Carbon Metabolism, Redox Status and Cellular Physiology in the Yeast *Saccharomyces cerevisiae* Devoid of *SOD1* or *SOD2* Gene

**DOI:** 10.3390/genes11070780

**Published:** 2020-07-11

**Authors:** Roman Maslanka, Renata Zadrag-Tecza, Magdalena Kwolek-Mirek

**Affiliations:** Department of Biochemistry and Cell Biology, Institute of Biology and Biotechnology, College of Natural Sciences, University of Rzeszow, 35-310 Rzeszow, Poland; romekmaslanka@gmail.com (R.M.); retecza@ur.edu.pl (R.Z.-T.)

**Keywords:** carbon metabolism, cell size, metabolic trade-off, pyridine nucleotide cofactors, reactive oxygen species, redox status

## Abstract

*Saccharomyces cerevisiae* yeast cells may generate energy both by fermentation and aerobic respiration, which are dependent on the type and availability of carbon sources. Cells adapt to changes in nutrient availability, which entails the specific costs and benefits of different types of metabolism but also may cause alteration in redox homeostasis, both by changes in reactive oxygen species (ROS) and in cellular reductant molecules contents. In this study, yeast cells devoid of the *SOD1* or *SOD2* gene and fermentative or respiratory conditions were used to unravel the connection between the type of metabolism and redox status of cells and also how this affects selected parameters of cellular physiology. The performed analysis provides an argument that the source of ROS depends on the type of metabolism and non-mitochondrial sources are an important pool of ROS in yeast cells, especially under fermentative metabolism. There is a strict interconnection between carbon metabolism and redox status, which in turn has an influence on the physiological efficiency of the cells. Furthermore, pyridine nucleotide cofactors play an important role in these relationships.

## 1. Introduction

Carbon metabolism has essential roles in cellular function and in the case of most cells is based on glucose, which is the primary source of energy. *Saccharomyces cerevisiae* yeast cells can utilise glucose, both by fermentation and aerobic respiration. However, they prefer the less energy efficient fermentative metabolism, even under aerobic conditions, which could be explained by the fact that glucose represses a number of genes that encode key respiratory enzymes [1]. If glucose concentration is very low, or yeast cells grow on non-fermentable carbon sources, respiratory metabolism can be exclusively observed. Carbon metabolism involves a network of interrelated pathways that provide energy in the form of adenosine triphosphate (ATP) molecules, precursors for biosynthesis of many cellular compounds and cofactors used in redox reactions. All of them are crucial for cell growth and proliferation but also are important for the maintenance of cellular redox homeostasis [2,3]. Cellular redox homeostasis is based on balance between generation and elimination of reactive oxygen species (ROS), which requires the presence of a well-coordinated system including proteins, low molecular weight compounds and pyridine nucleotide cofactors. Since Jensen reported his results, mitochondrial respiration is considered the major cellular source of ROS [4]. However, recently, more and more studies underline the existence of non-mitochondrial ROS sources within the cell [5,6,7]. In general, non-mitochondrial ROS are quite well characterized in human cells [8], but there are also studies presenting the possibility of the existence of non-mitochondrial ROS sources in the case of yeast cells [5,6,7,9]. Among non-mitochondrial ROS sources, reduced nicotinamide adenine dinucleotide phosphate (NADPH) oxidases appear to be one of the most important [9,10,11] as they catalyse the production of superoxide anion from oxygen and NADPH [8]. It is worth noting that recent studies identified the existence and importance of NADPH oxidase 1 (Yno1p) also in yeast cells [9,12]. Maintenance of redox balance requires efficient reducing processes in which, besides proper glutathione level, a balance between pyridine nucleotide pairs plays a significant role [2,13,14,15]. The NAD^+^/NADH couple is primarily responsible for oxidation reactions and has an impact on the level of catabolic reactions in the cell. In turn, the NADPH/NADP^+^ couple is responsible for reduction reactions [16]. NADPH is associated with cellular protection against oxidative stress, mainly due to its role in regeneration of reduced glutathione (GSH) [13,17] but also due to its reactive oxygen species (ROS) scavenging capabilities [18]. Furthermore, NADPH has an impact on the level of anabolic reactions and is an essential intracellular reductant used inter alia in biosynthesis of amino acids, lipids and nucleotides [2,17]. A strict connection between NADPH and cellular biosynthesis is further confirmed by way of NADPH formation. During fermentation, NADPH is mainly produced by oxidative steps of the pentose phosphate pathway (PP pathway). This route of glucose utilisation is generally considered as a source of intermediates for aromatic amino acids and nucleotides biosynthesis [19,20]. In turn, the biosynthetic processes have an impact on cell size and thus on cell proliferation. Cell growth and proliferation are highly energy-consuming processes [21], and this is the reason why cells have to adapt to changes in nutrient availability, which may cause the existence of metabolic trade-off between cellular metabolic pathways [22,23]. Such cellular interconnection between carbon metabolism, redox homeostasis and physiological parameters of the cell are crucial for proper cell function and its adaptation to environmental fluctuations.

The studies based on glucose metabolism and respiratory-deficient mutant strains and concerning the linkage between carbon metabolism and ROS generation provided support for the hypothesis that non-mitochondrial ROS sources do exist in yeast cells [5,7]. However, the issue of non-mitochondrial ROS sources in yeast cells is relatively new and requires further clarification. Therefore, the aim of this study was to explore the connection between the type of metabolism and redox status of the cell and their influence on selected parameters of cellular physiology in conditions where the antioxidant defense is defective. For that purpose, we used yeast strains devoid of superoxide dismutase (SOD), the main antioxidant enzyme in cells. In *S. cerevisiae* yeast there are two SOD isoenzymes that differ in terms of sub-cellular localization: a copper-zinc superoxide dismutase (Sod1p), which is located in the cytosol and in the intermembrane space of mitochondria, and manganese-superoxide dismutase (Sod2p), which is located in the mitochondrial matrix [24]. In addition, we have introduced a new experimental approach. Most studies concerning carbon metabolism and ROS generation are usually focused on comparing yeast cells cultivated in fermentative or respiratory conditions or in conditions with the fermentable and non-fermentable carbon sources [5,25]. Our experimental approach differentiates the fermentative and respiratory conditions through modulation of the mitochondrial activity and involves: medium with glycerol as a non-fermentable source of carbon—respiratory conditions; medium with 0.5% glucose (calorie restriction)—respiro-fermentative conditions; medium with 2% glucose (calorie optimum) and medium with 4% glucose (calorie excess)—fermentative conditions. These varied conditions are designed on the one hand to show the scale of the adaptive capabilities of the cell and on the other hand to provide new arguments for the existence of non-mitochondrial ROS sources in yeast cells.

## 2. Materials and Methods

### 2.1. Chemicals

Dihydroethidium (DHET) and rhodamine B hexyl ester were from Molecular Probes (Eugene, OR, USA). BacTiter-Glo Microbial Cell Viability Assay, NAD/NADH-Glo Assay and NADP/NADPH-Glo Assay kits were from Promega (Madison, WI, USA). Coomassie Protein Assay Reagent was from Thermo Scientific (Waltham, MA, USA). All other reagents were purchased from Sigma-Aldrich (Poznan, Poland). Components of culture media were from BD Difco (Becton Dickinson and Company, Spark, USA) except for glucose (POCH, Gliwice, Poland).

### 2.2. Yeast Strains and Growth Conditions

The following yeast strains were used: wild-type (WT) BY4741 MATa his3 leu2 met15 ura3 and two mutant strains isogenic to BY4741: Δ*sod1* and Δ*sod2* (EUROSCARF, Scientific Research and Development GmbH, Oberursel, Germany). Yeast was grown in liquid YP medium (1% yeast extract, 1% yeast bacto-peptone) with 3% glycerol or with different glucose concentrations (0.5, 2 and 4%) on a rotary shaker at 150 rpm, at 28 °C. The time of yeast cell cultivation to obtain the exponential phase of growth and maintain a specific type of metabolism (respiro-fermentative or fermentative) depended on the glucose concentration in the medium and was adjusted as described by [7]. Considering the low rate of growth of yeast cells in medium with glycerol, the time of cultivation in that case was appropriately longer.

### 2.3. Assessment of Cellular ATP Content

The level of ATP in the yeast cells was determined with BacTiter-Glo Microbial Cell Viability Assay according to the manufacturer’s protocol (Promega, Madison, WI, USA) with our own modifications. Cells from the early exponential phase of growth were suspended in a 100 mM phosphate buffer with pH 7.0, containing 0.1% glucose and 1 mM EDTA. A sample of the cell suspension with a density of 10^6^ cells/ml was used for determination purposes. The luminescent signal, proportional to the amount of ATP, was recorded using an Infinite 200 microplate reader (Tecan Group Ltd., Männedorf, Switzerland), once the luminescence signal had achieved a stable value.

### 2.4. Estimation of Mitochondrial Network Morphology

Morphology of the mitochondrial network was determined using rhodamine B hexyl ester, whose fluorescence depends on mitochondrial membrane potential. Cells from the early exponential phase of growth were washed twice with sterile water and suspended in a 10 mM HEPES buffer with pH 7.4, containing 5% glucose. Incubation with 100 nM rhodamine B was conducted for 20 min in the dark at 28 °C. After, incubation mitochondrial network was visualised using fluorescence microscopy at λex = 555 nm and λem = 579 nm. The microscopic images, which present typical results from one of two experiments, were captured with a BX-51 microscope equipped with a DP-72 digital camera (Olympus, Tokyo, Japan) and cellSens Dimension v1.0 software.

### 2.5. Determination of ROS Content

The level of reactive oxygen species was assessed with dihydroethidium (DHET; 10.7 µM final concentration; stock solution in DMSO) and 2′,7′-dichlorodihydrofluorescein diacetate (H_2_DCF-DA; 15.8 µM final concentration; stock solution in ethanol). DHET is oxidised by superoxide anion to fluorescent hydroxyethidium, but also undergoes a non-specific oxidation to form ethidium in the presence of other oxidants, e.g., hydroxyl radical, peroxynitrite and perferryl iron [26,27]. In turn, H_2_DCF-DA after entering the cell is hydrolysed with release of 2′,7′-dichlorodihydrofluorescein (H_2_DCF) and then it is oxidised to fluorescent 2′,7′-dichlorofluorescein (DCF) by intracellular oxidants, the formation which is caused by changes in iron or haem signaling or peroxynitrite generation [26,27]. Yeast cells from the early exponential phase of growth were washed twice with sterile water and suspended to a final density of 10^8^ cells/mL in a 100 mM phosphate buffer with pH 7.0, containing 0.1% glucose and 1 mM EDTA. DHET or H_2_DCF-DA was added to 200 µL of cell suspension and the fluorescence measurement was immediately performed. The increase in the kinetics of the fluorescence, due to oxidation of the fluorogenic probes, was measured using an Infinite 200 microplate reader (Tecan Group Ltd., Männedorf, Switzerland). Measurements were performed for 30 min at 28 °C at λex = 518 nm, λem = 605 nm for DHET and λex = 495 nm, λem = 525 nm for H_2_DCF-DA. ROS content was expressed as a relative rate of fluorescence increase.

### 2.6. Protein Extraction

The cells from the early exponential phase of growth were washed twice with sterile water and suspended in cold homogenization buffer (20 mM phosphate buffer with pH 7.0, containing 1 mM EDTA and 1 mM PMSF). The cells were disrupted with 0.5 mm glass beads, vortexed in 7 cycles for 30 s with intervals for cooling the sample on ice and then centrifuged (14,000× *g*, 15 min, 4 °C). Supernatants were transferred to fresh tubes and immediately frozen at −80 °C. Protein concentration was determined using Coomassie Protein Assay Reagent (Thermo Scientific, Waltham, MA, USA).

### 2.7. NADPH Oxidase Assay

NADPH oxidase activity was measured using a lucigenin-enhanced chemiluminescence method as described previously [28] with our own modification. Measurements of the increase in the kinetics of the luminescence were performed every 30 s for 15 min at 28 °C using an Infinite 200 microplate reader (Tecan Group Ltd., Männedorf, Switzerland). The reaction was initiated by addition of 20 µL of cell extract (2 mg protein per ml) to the assay buffer (50 mM phosphate buffer with pH 7.0, containing 1 mM EGTA, 150 mM sucrose, 1 mM CaCl_2_, 5 µM lucigenin and 200 µM NADPH). There was no measurable activity in the absence of NADPH. Activity was expressed as a relative rate of luminescence increase.

### 2.8. Determination of Thiol Groups Content

The content of thiol groups in the cell extracts was estimated with 5,5′-dithiobis-(2-nitrobenzoic acid) (DTNB; 10 mg/mL stock solution in 0.5% NaCO_3_) in 300 mM Tris-HCl buffer with pH 8.2 containing 1 mM EDTA. Absorbance was measured after 20 min of incubation in the dark, at room temperature, using an Infinite 200 microplate reader (Tecan Group Ltd., Männedorf, Switzerland), at λ = 412 nm against a reagent blank. The thiol groups concentration was calculated using ε_412 nm_ = 13.6 mmol^−1^ l cm^−1^ and expressed in nmol per mg of protein.

### 2.9. Determination of Tryptophan Content

The tryptophan content in the cell extract (0.1 mg protein per mL) was estimated as described previously [29] using an Infinite 200 microplate reader (Tecan Group Ltd., Männedorf, Switzerland), at λex = 290 nm, λem = 325 nm. The results were expressed in arbitrary units.

### 2.10. Determination of Cell Growth

Liquid yeast cultures were cultivated in a Titramax 1000 incubator (Heidolph Insruments GmbH & CO. KG, Schwabach, Germany) with shaking at 28 °C. Growth was monitored turbidimetrically at λ = 600 nm using an Anthos 2010 type 17 550 microplate reader (Anthos Labtec Instruments, Salzburg, Austria). Measurements were performed at 1 h intervals for 12 h and after 22 h of cultivation in the case of medium with different glucose concentrations, and at 1 h intervals for 22 h in the case of medium with 3% glycerol.

For spotting test, yeast exponential phase cultures were diluted to give suspensions of 10^7^, 10^6^, 10^5^ and 10^4^ cells/mL. Aliquots of 5 µL of each suspension were inoculated on appropriate solid YP medium with 3% glycerol or with different glucose concentrations. Colony growth was inspected after 48 h in the case of medium with different glucose concentrations and after 72 h in the case of medium with 3% glycerol.

### 2.11. Estimation of Cell Size

Cell size in the population was estimated through analysis of microscopic images captured with a BX-51 microscope equipped with a DP-72 digital camera (Olympus, Tokyo, Japan). Cell diameter was measured in two perpendicular planes for each cell using cellSens Dimension v1.0 software. For each yeast strain cultivated in the liquid YP medium with 3% glycerol or with different glucose concentrations, at least 300 cells were counted. The results were presented as a histogram, which is a graphic representation of cell size distribution.

### 2.12. Assessment of Pyridine Nucleotide Cofactors Content

The NAD(H) and NADP(H) content in the yeast cells was assessed with NAD/NADH-Glo Assay and NADP/NADPH-Glo Assay kits according to the manufacturer’s protocols (Promega, Madison, WI, USA). In the presence of NAD(H) and NADP(H), a reductase provided with the assay reduces the proluciferin reductase substrate to form luciferin. Luciferin is then quantified using Ultra-Glo Recombinant Luciferase, and the produced light signal is proportional to the amount of NAD^+^ or NADH and NADP^+^ or NADPH in the sample. The BY4741 cells from the early exponential phase of growth were centrifuged, washed, suspended to a density of 2 × 10^6^ cells/mL in PBS buffer and used for determination purposes. Luminescence was recorded for 3 h using an Infinite 200 microplate reader (Tecan Group Ltd., Männedorf, Switzerland). The value of the blank was subtracted each time. The results were presented as individual pyridine nucleotide cofactors content. In addition, NAD^+^/NADH and NADPH/NADP^+^ ratios were shown.

### 2.13. Statistical Analysis

The results are presented as mean ± SD from at least three independent experiments. The statistical analysis was performed using IBM SPSS 21.0 software. The statistical significance of the differences between the wild-type strain in comparison to the mutant strains were estimated using one-way ANOVA with the Dunnett’s post hoc test. The statistical significance of the differences between means of the four media compared was evaluated using one-way ANOVA with the Tukey post-hoc test. Homogeneity of variance was checked using Levene’s test. The values were considered significant at *p* < 0.05. Used designation: differences between strains * *p* < 0.05, ** *p* < 0.01, *** *p* < 0.001; differences between media a—different from medium with 3% glycerol, b—different from medium with 0.5% glucose, c—different from medium with 2% glucose, d—different from medium with 4% glucose.

## 3. Results

### 3.1. Type of Metabolism Determines the ATP Content and Mitochondrial Network Morphology

**Figure 1:**
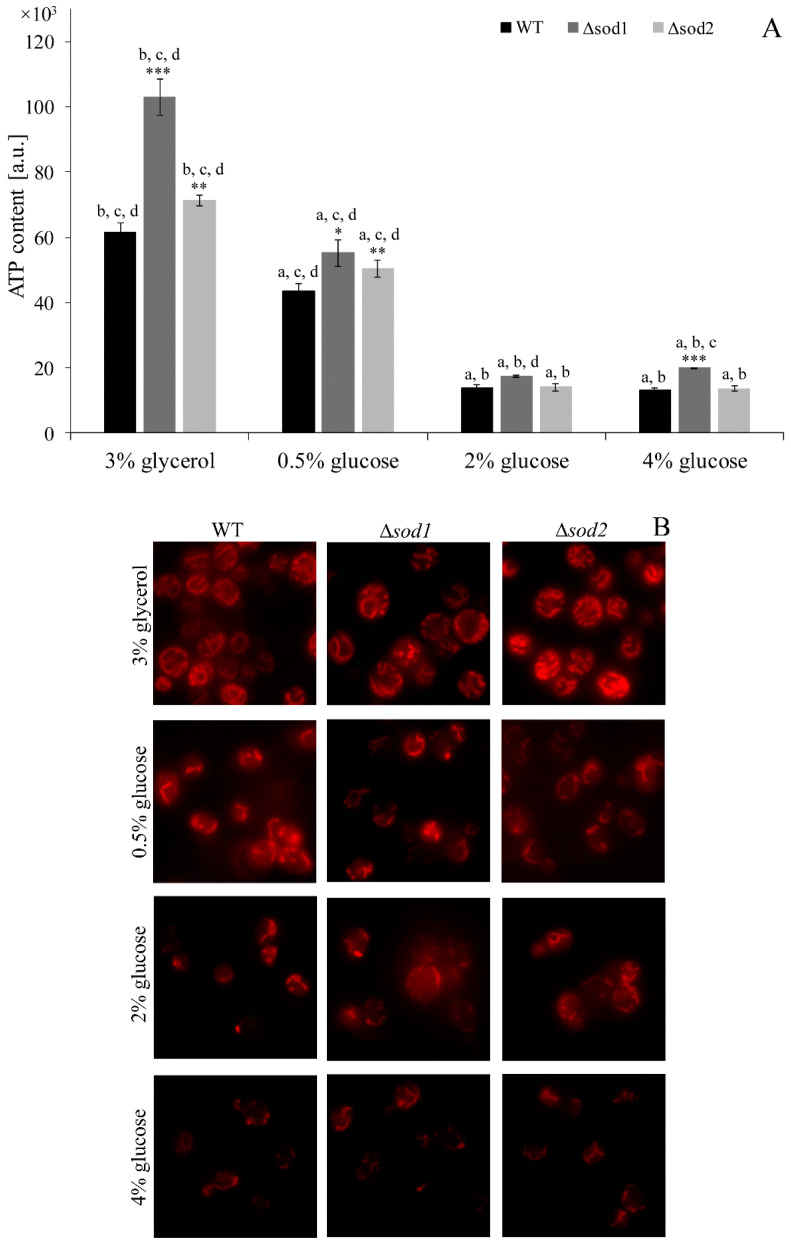
The adenosine triphosphate (ATP) content (**A**) and mitochondrial network morphology (**B**) in the wild-type (WT) strain, Δ*sod1* and Δ*sod2* mutants cultivated in medium with 3% glycerol or different glucose concentration. Data are presented as mean ± SD from three independent experiments. * *p* < 0.05, ** *p* < 0.01, *** *p* < 0.001 as compared to the WT strain; a—different from medium with 3% glycerol, b—different from medium with 0.5% glucose, c—different from medium with 2% glucose, d—different from medium with 4% glucose. The microscopic images present the typical results of the duplicate experiment.

Aerobic respiration that occurs in the mitochondria provides the cells with a large amount of energy. It was confirmed in this study that in the yeast cells cultivated in the medium with glycerol (non-fermentable source of carbon) the level of ATP was very high (Figure 1A) and the mitochondrial network was highly developed (Figure 1B). However, it is worth noting that the ATP content was about 40% and 10% higher in the Δ*sod1* and Δ*sod2* mutants, respectively, in comparison to the WT strain (Figure 1A). The mitochondrial network was well extended also in the case of yeast cells cultivated in the medium with 0.5% glucose where energy was produced by both fermentation and respiration (a mix of both) (Figure 1B). However, the ATP content in the WT cells cultivated in these conditions was 30% lower in comparison to the WT cells cultivated in the medium with glycerol (Figure 1A). The level of ATP in the Δ*sod1* and Δ*sod2* mutants was higher by about 27% and 16%, respectively, in comparison to the WT strain (Figure 1A). On the other hand, when the cells produced energy only in the fermentation process (the yeast cells were cultivated in the medium with 2% or 4% glucose) the mitochondrial network was poorly developed (Figure 1B). The ATP content in the WT cells cultivated in these conditions was four-fold lower in comparison to the same strain cells cultivated in the medium with glycerol (Figure 1A). In addition, a higher level of ATP was found in the Δ*sod1* mutant and no differences in the Δ*sod2* mutant, in both cases in comparison to the WT strain (Figure 1A). These results confirm that the ATP content and mitochondrial network morphology strictly depend on the type of metabolism of the yeast cells, but what is more important, deletion of *SOD2* and especially *SOD1* increases the mitochondrial activity and thus ATP formation.

### 3.2. Intracellular ROS Content Depends on the Type of Metabolism

**Figure 2:**
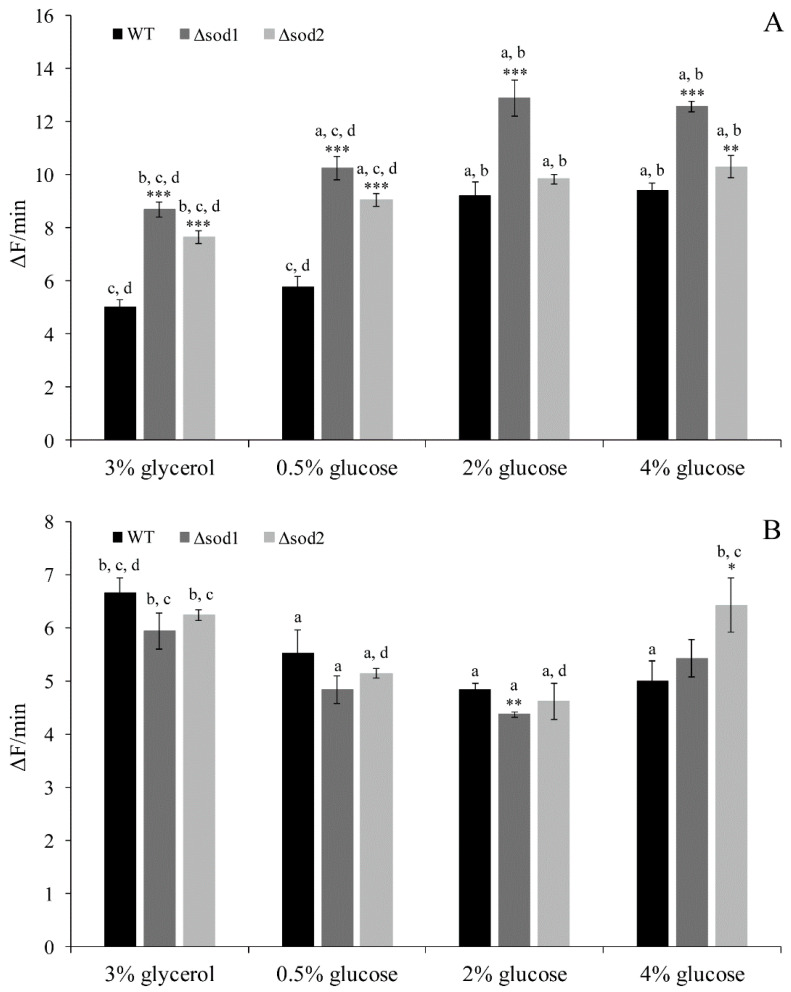
The reactive oxygen species (ROS) content in the WT strain, Δ*sod1* and Δ*sod2* mutants cultivated in medium with 3% glycerol or different glucose concentration was estimated by the rate of fluorescence increase due to dihydroethidium (DHET) (**A**) and 2’,7’-dichlorodihydrofluorescein (H_2_DCF) (**B**) oxidation within cells. Data are presented as mean ± SD from three independent experiments. * *p* < 0.05, ** *p* < 0.01, *** *p* < 0.001 as compared to the WT strain; a—different from medium with 3% glycerol, b—different from medium with 0.5% glucose, c—different from medium with 2% glucose, d—different from medium with 4% glucose.

Cell metabolism causes the formation of ROS under physiological conditions. The level of ROS was estimated with DHET, which is oxidised by superoxide anion to fluorescent hydroxyethidium and non-specific by hydroxyl radical, peroxynitrite and perferryl iron to fluorescent ethidium [26,27]. In the WT strain the lowest level of ROS was observed in cells cultivated in the medium with glycerol (Figure 2A). A slightly higher level was observed in the case of 0.5% glucose medium, and it was approx. 40% higher in the case of 2% and 4% glucose medium. The ROS content in the WT cells cultivated in the medium with 2% and 4% glucose was at the same level (Figure 2A). A similar relationship was observed for both the Δ*sod1* and Δ*sod2* mutants (Figure 2A). However, the level of ROS in the Δ*sod1* mutant was significantly higher for all tested culture media in comparison with the WT strain (Figure 2A). A higher level of ROS than in the WT strain was observed also in the case of the Δ*sod2* mutant, but this effect was much more pronounced in the medium with glycerol and 0.5% glucose in comparison to the medium with 2% or 4% glucose (Figure 2A). The level of ROS in the Δ*sod2* mutant was lower than in the Δ*sod1* mutant for all tested culture media (Figure 2A). These results confirm the essential role of SOD1 in reducing the level of ROS (superoxide anion) in cells. They also confirm the importance of SOD2, especially when cells produce energy solely through aerobic respiration (medium with glycerol) or both fermentation and respiration (medium with 0.5% glucose).

In turn, when ROS content was estimated with H_2_DCF-DA, other relationships than in the case of DHET were observed (Figure 2A,B). This was due to the fact that the probe is not substrate specific and may be used as a redox indicator detecting intracellular oxidant formation caused by changes in iron or haem signaling or peroxynitrite generation [26,27]. In the WT strain the highest level of ROS was observed in cells cultivated in the medium with glycerol (Figure 2B). In the case of 0.5% and 2% glucose, an approximately 17% and 27% lower level of ROS was observed, respectively, in comparison to cells cultivated in the medium with glycerol. The ROS content in the WT strain cultivated in the medium with 2% and 4% glucose was at the same level (Figure 2B). A similar relationship was observed for both the Δ*sod1* and Δ*sod2* mutants, but only in the case of the medium with glycerol and 0.5% and 2% glucose (Figure 2B). In turn, when the Δ*sod2* mutant was cultivated in the medium with 4% glucose, an increase in ROS level was demonstrated. The level of ROS in the case of 4% glucose was similar to the level achieved for the medium with glycerol (Figure 2B). These results confirm a higher intracellular level of ROS during aerobic respiration compared to fermentation.

It is worth mentioning here that, besides the mitochondrial, there are also non-mitochondrial sources of ROS in the cells. A non-mitochondrial source of superoxide anion may be among other NADPH oxidase 1 (Yno1p). It was demonstrated in this study that this enzyme is active in the yeast cells, but its activity does not depend on the type of metabolism (a similar level was observed both when the cells were cultivated in the medium with glycerol and glucose).

### 3.3. Thiol Groups Content Depends on the Type of Metabolism

**Figure 3:**
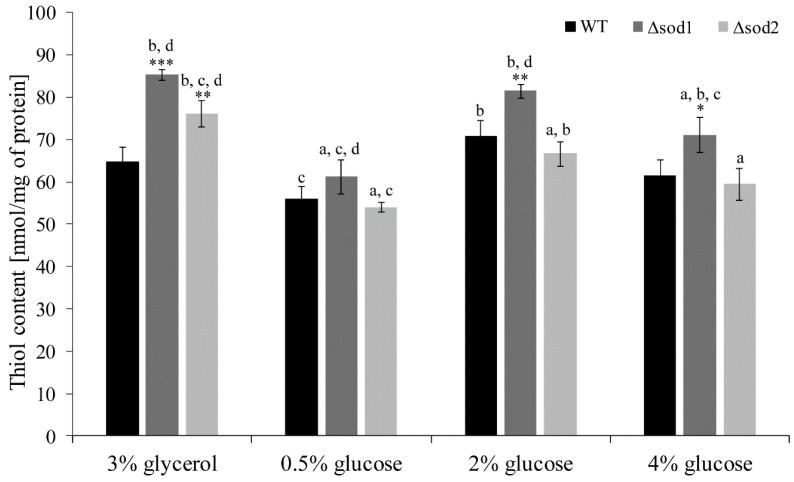
The thiol groups content in the WT strain, Δ*sod1* and Δ*sod2* mutants cultivated in medium with 3% glycerol or different glucose concentration. Data are presented as mean ± SD from three independent experiments. * *p* < 0.05, ** *p* < 0.01, *** *p* < 0.001 as compared to the WT strain; a—different from medium with 3% glycerol, b—different from medium with 0.5% glucose, c—different from medium with 2% glucose, d—different from medium with 4% glucose.

The level of thiol groups (included both in proteins and glutathione) is important information about intracellular redox status. It was demonstrated that the thiol groups content in the WT cells cultivated in the medium with glycerol and 2% and 4% glucose was at a similar level (Figure 3). A slightly lower level was observed in the case of 0.5% glucose medium (Figure 3). In turn, the level of thiol groups in the Δ*sod1* mutant was significantly higher than in the WT strain in the case of all tested culture media, except 0.5% glucose. This effect was much more pronounced in the medium with glycerol in comparison to the medium with 2% and 4% glucose (Figure 3). The higher level of thiol groups was observed also in the case of Δ*sod2* mutant and medium with glycerol, but this level was lower than in the Δ*sod1* mutant (Figure 3). This difference was not observed in the case of medium with 0.5%, 2% and 4% glucose (Figure 3). These results show that the type of metabolism may influence the thiol groups content in the cells, which is particularly evident in the case of the Δ*sod2* mutant and under respiratory metabolism.

### 3.4. Type of Metabolism Determines the Cell Population Growth

**Figure 4:**
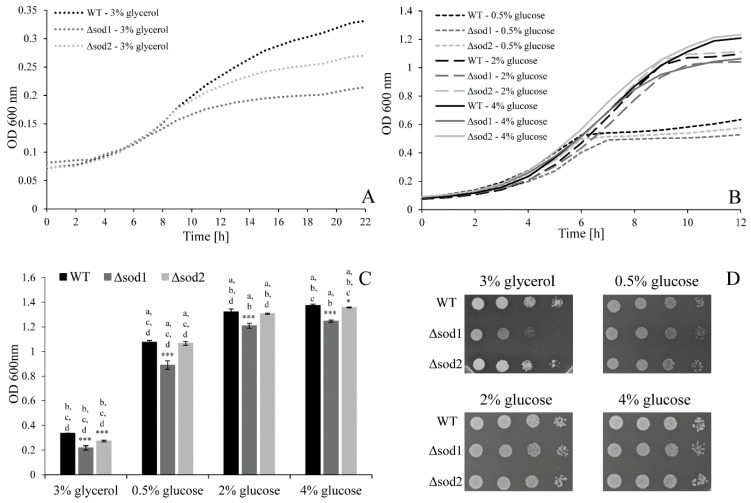
The growth of the WT strain, Δ*sod1* and Δ*sod2* mutants on both liquid and solid medium with 3% glycerol or different glucose concentration. Kinetics of growth was monitored turbidimetrically every 1 h for 22 h for medium with 3% glycerol (**A**), every 1 h for 12 h for medium with different glucose concentration (**B**), and after 22 h (**C**). Data are presented as mean ± SD from three independent experiments. * *p* < 0.05, *** *p* < 0.001 as compared to the WT strain; a—different from medium with 3% glycerol, b—different from medium with 0.5% glucose, c—different from medium with 2% glucose, d—different from medium with 4% glucose. In the spotting test (**D**) colony growth was recorded after 72 h for medium with 3% glycerol and after 48 h for medium with different glucose concentrations. Successive spots initially contained 50,000, 5000, 500 and 50 cells.

Cultivation of yeast cells in the medium with glycerol and with different glucose concentrations resulted in different time periods for reaching the growth curve plateau level (Figure 4A,B). The plateau level was reached after 6 h for both the WT and Δ*sod2* strains and after 7 h for the Δ*sod1* mutant in the medium with 0.5% glucose; it was reached after 9 and 10 h in the medium with 2% glucose and after 11 and 9 h in the medium with 4% glucose for both the WT and Δ*sod2* strains and for the Δ*sod1* mutant, respectively. The shapes of growth curves in media with 2% and 4% glucose were similar but after 12 h the cultures reached different maximum optical density (OD) values (Figure 4B). In turn, when the cells were cultivated in the medium with glycerol, the plateau level was reached after 10‒11 h for the Δ*sod1* mutant, after 12 h for the Δ*sod2* mutant and after 15 h for the WT strain (Figure 4A). The influence of type of metabolism on the level of cell growth was compared also after 22 h of cultivation (Figure 4C). The lowest OD values were observed in the case of the medium with glycerol. Higher OD values were associated with increased glucose concentrations in the medium. The growth of the Δ*sod1* mutant was significantly weaker in comparison to the WT strain for all tested culture media (Figure 4C). On the other hand, lower OD values for the Δ*sod2* mutant compared to the WT strain were observed only in the case of growth in the medium with glycerol and 4% glucose (Figure 4C). On solid medium, the growth of the Δ*sod1* and Δ*sod2* mutants was weaker in comparison to the WT strain only in the case of medium with glycerol (Figure 4D). These results confirm that the lack of SOD1 causes a reduction of cell population growth, especially in the medium with glycerol. It may be associated with increased iron demand, which is necessary to reconstitute Fe-S cluster-containing enzymes that are inactivated in the condition of excess superoxide [30]. In turn, lack of SOD2 causes a reduction of cell population growth in media with glycerol and 4% glucose, which may be a result of a high level of ROS in the Δ*sod2* mutant cells in these conditions (Figure 2B).

### 3.5. Type of Metabolism Determines the Cell Size

**Figure 5:**
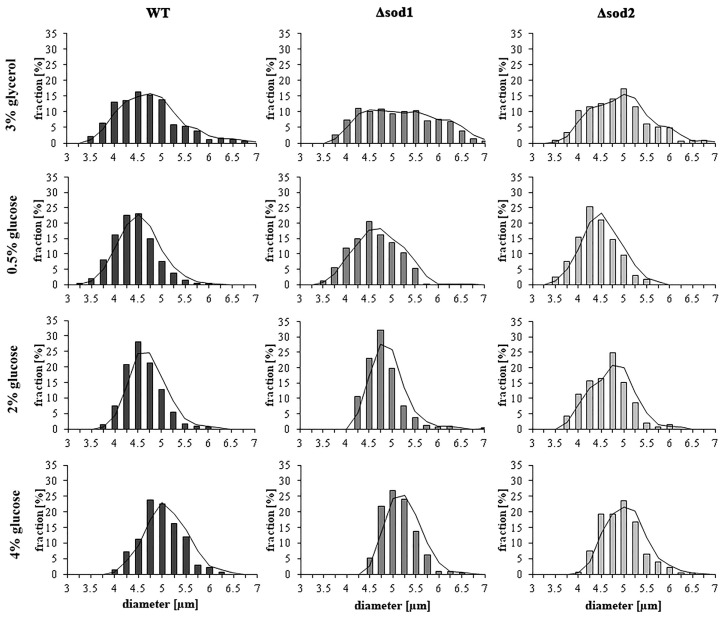
The cell size of the WT strain, Δ*sod1* and Δ*sod2* mutants cultivated on medium with 3% glycerol or different glucose concentrations. Diameter of the cells was estimated through analysis of microscopic images using Olympus cellSens Dimension software. The results are presented as a histogram; *n* = 300 cells.

Glucose is an important molecule for the biosynthesis process, which has a significant impact on the size of the yeast cells in a population. In our previous study, we showed that there is a relationship between cell size and extracellular glucose concentration. The mean value of cell size increased with increasing extracellular glucose concentration [7]. This study confirms such a relationship for all tested strains (Figure 5). Increase in the mean cell size is represented by changes in distribution of cell diameters in the population, which is shown in the histogram (Figure 5). The dominant fraction of the cell population is shifted towards a higher size. The centre of cell diameter dispersion in the case of the WT strain cultivated in the medium with 0.5% glucose was 4‒4.5 µm. This parameter was 4.25‒4.75 µm and 4.5‒5 µm in the medium with 2% and 4% glucose, respectively (Figure 5). There were also changes in the number of cells with the lowest size (<4 µm). They represented 25, 9 and 1.5% of the cell population in the case of 0.5%, 2% and 4% glucose medium, respectively (Figure 5). In turn, the number of cells with the highest size (>5.5 µm) increased with glucose concentration. They represented 0.5%, 1.5% and 5.5% of the cell population in the case of 0.5%, 2% and 4% glucose medium, respectively (Figure 5). A similar relationship was observed for both the Δ*sod1* and Δ*sod2* mutants (Figure 5). However, the Δ*sod1* mutant shows increased cell size for all tested culture media, in comparison to the WT strain (Figure 5). On the other hand, in the medium with glycerol a wide differentiation of yeast cell sizes in the population was noted (determination of dominant fraction is difficult) (Figure 5). These results confirm that the type of metabolism influences the size of yeast cells.

### 3.6. Pyridine Nucleotide Cofactors Content Depends on the Type of Metabolism

**Figure 6:**
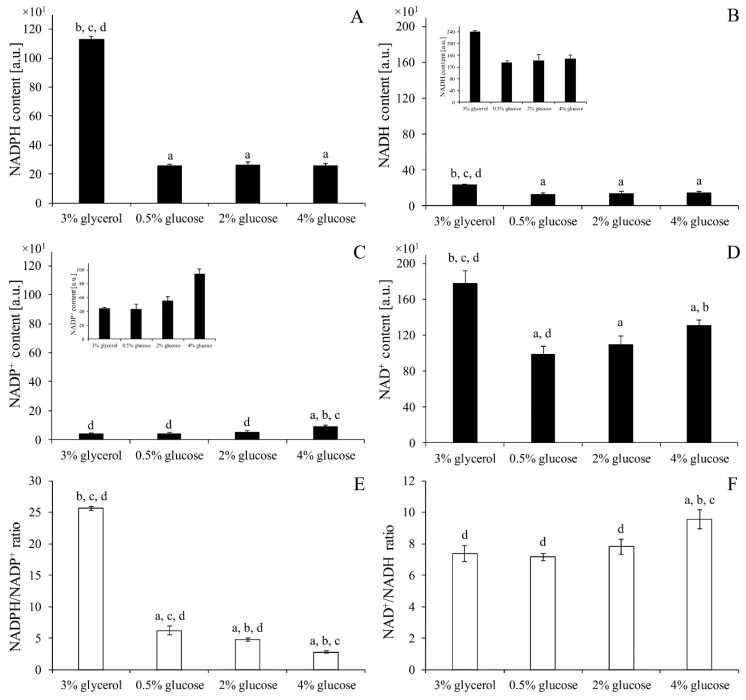
The content of individual pyridine nucleotide cofactors NADPH (**A**), NADH (**B**), NADP^+^ (**C**), NAD^+^ (**D**) was assessed in the cells WT strain cultivated in medium with 3% glycerol or different glucose concentration. NADPH/NADP^+^ (**E**) and NAD^+^/NADH (**F**) ratios were also calculated. Data are presented as mean ± SD from three independent experiments. a—different from medium with 3% glycerol, b—different from medium with 0.5% glucose, c—different from medium with 2% glucose, d—different from medium with 4% glucose.

NAD(H) and NADP(H) content depends on the source of carbon and its content in the culture medium. To facilitate analysis, this relationship was examined only for the WT strain. The highest levels of both NADPH and NADH were observed in the cells cultivated in the medium with glycerol (Figure 6A,B). These cofactors were at the same level in the cells cultivated in the medium with 0.5%, 2% or 4% glucose (Figure 6A,B). On the other hand, the level of NADP^+^ was the highest in the medium with 4% glucose. The NADP^+^ content did not differ in the case of other tested culture media (Figure 6C). In turn, the level of NAD^+^ was the highest in the case of the medium with glycerol, lower in the medium with 4% glucose and the lowest in the medium with 0.5% glucose (Figure 6D). As important as the content of individual pyridine nucleotide cofactors in cells is the relationship between them. The NADPH/NADP^+^ ratio was the highest in the cells cultivated in the medium with glycerol, lower in the medium with 0.5% glucose, even lower in the medium with 2% glucose and the lowest in the 4% glucose (Figure 6E). The NAD^+^/NADH ratio was different only in the case of medium with 4% glucose. This ratio for other tested culture media was at a similar level, which was lower in comparison to the cells cultivated in the medium with 4% glucose (Figure 6F). These results confirm that both the level of pyridine nucleotide cofactors and the ratio between them adjust to metabolic changes and current cellular demands.

### 3.7. Pyridine Nucleotide Cofactors Content Corresponds to Tryptophan Content

Pyridine nucleotide cofactor production requires compounds for its synthesis. The basic form from pyridine nucleotide cofactors is NAD^+^, which can be further converted to NADH, NADP^+^ and NADPH via dehydrogenases, ATP-NAD kinases and ATP-NADH kinases, respectively. NAD^+^ is synthesized de novo from tryptophan by its conversion in the kynurenine pathway. The results demonstrated that cells cultivated in the medium with glycerol had a significantly increased level of tryptophan in comparison to cells cultivated in the medium with glucose, independent of glucose concentration (Figure S1). These results show that the higher level of pyridine nucleotide cofactors observed in the cells cultivated in the medium with glycerol may be a result of its higher de novo formation from tryptophan [31].

## 4. Discussion

From a biophysical point of view, cells are open thermodynamic systems that constantly exchange energy and matter with the environment [32]. Therefore, cells must respond and adapt to changes in the environment. The availability of nutrients is a powerful factor influencing metabolic processes, which in the case of *Saccharomyces cerevisiae* yeast is manifested by the choice between fermentation or respiration as a result of the specific metabolic strategy associated with a balance of gains and losses [23,33]. Metabolic pathways of carbon utilisation are associated with generation of ROS. Respiratory chain reactions taking place in the mitochondria were considered the major cellular sources of ROS [4]. In yeast the relationship between ROS generation, mitochondria activity and types of metabolism have been studied thoroughly [7,34,35,36,37]. Our previous work implies that calorie excess has a negative impact on cell physiology, inter alia by increasing the ROS content, which was independent from the mitochondrial activity [7]. The results presented in this work also show that non-mitochondrial sources are an important pool of ROS in yeast cells, especially under fermentative metabolism. On the other hand, the generation of ROS in the case of oxidative respiration conditions shows that the respiratory chain is still an important part of ROS sources (Figure 2). It means that the source of ROS depends on the type of metabolism and is confirmed among others by the results of ROS content in the case of the Δ*sod2* mutant. It has been observed that disruption of the *SOD2* gene has significant impact on the level of ROS generated during respiratory metabolism (Figure 2). In turn, disruption of the *SOD1* gene causes disorder in the elimination of ROS generated from both mitochondrial and non-mitochondrial sources (Figure 2). As was shown here, as well as in the previous work [7], fermentative metabolism triggered by high levels of glucose is connected mainly with a high level of superoxide anion generation (Figure 2A). It was observed also in the case of mammalian cell cultures and on *C. elegans* [38,39,40]. An elevated level of superoxide anion connected with glucose concentration is also observed in other studies which used *S. cerevisiae* [41] and *Sc. pombe* [42] yeast. The results of those studies suggest that negative aspects of high glucose concentration may be connected with an increased activity of the cAMP/PKA signaling pathway [41,42]. Studies show that an active cAMP/PKA pathway on the one hand promotes cellular protein biosynthesis by inducing ribosome biogenesis, while on the other hand, it reduces the stress response by suppressing expression of Msn2/4-mediated genes [43]. These two issues can be taken into account to explain the higher level of ROS observed under 2% and 4% glucose concentration (Figure 2A). A high level of protein biosynthesis enforces an increased rate of protein folding that takes place in the endoplasmatic reticulum (ER), during which ROS are formed [44]. Connection between ER, biosynthesis and ROS production is further strengthened by studies demonstrating the existence of Yno1p, the only NADPH oxidase discovered so far in yeast [9]. The studies performed on mammalian cells indicate that NADPH oxidase is considered as an important non-mitochondrial source of ROS. This ER-localised enzyme produces superoxide by the one-electron reduction of oxygen in a NADPH-dependent fashion [9,10,45,46]. The issue of NADPH oxidase activity in the case of yeast cells requires further analysis, but it is worth noting that despite the lack of differences in its activity between conditions with different carbon sources (as demonstrated in this study), the sole fact that this enzyme is active might point to its role in non-mitochondrial ROS production. High levels of ROS observed in our research under 2% and 4% glucose concentration (Figure 2A) may also be connected with changes in the cell stress response, since the Msn2p and Msn4p transcriptional factors exhibit a cytoplasmic location in the presence of glucose. Hence, expression of genes encoding antioxidant proteins or enzymes involved in proteolysis is repressed [43,47].

Besides controlling the production of ROS, maintenance of intracellular redox balance also requires efficient reducing processes in which pyridine nucleotide cofactors plays a significant role. In this study, we observed that the level and ratio of pyridine nucleotide cofactors differ between type of carbon source (glucose/glycerol) and also within glucose concentrations. Both NADPH/NADP^+^ and NAD^+^/NADH ratios (Figure 6E,F) may suggest that when cells carry out fermentative metabolism the intracellular environment is more oxidative than under aerobic respiration. This is somewhat surprising, considering that the cAMP/PKA pathway stimulates both glycolytic and biosynthetic flux with simultaneous inhibition of respiratory metabolism. However, it should be highlighted that the amount of pyridine nucleotide cofactors does not remain at steady state: the cofactors are continually formed and used depending on the actual demands of the cell. For that reason, the ratio of pyridine nucleotide cofactors, should not be used as a sole indicator of the cell redox state but in combination with other parameters, e.g., content of individual cofactors. Higher levels of both NAD^+^ and NADH observed in cells cultivated in glycerol are strictly connected with metabolism based on aerobic respiration (Figure 6B,D). Under respiratory metabolism NADH is produced not only in a glycolysis pathway but also in the citric acid cycle and is subsequently oxidised back to NAD^+^ in oxidative phosphorylation. The relation between NAD^+^ and NADH under conditions of different glucose concentrations is more complex. Studies analyzing changes in NAD^+^ concentration are ongoing and show that the basal levels of NAD^+^ result from a dynamic balance between NAD^+^ synthesis and NAD^+^ consumption [48]. The literature data which show increased yeast lifespan in calorie restriction conditions in general connected this outcome with an increase in the NAD^+^/NADH ratio [49,50], although there are also results showing unchanged NAD^+^ levels and NAD^+^/NADH ratio and are independent from their lifespan extension [51]. In this study the similar values of NAD^+^ and NAD^+^/NADH ratio were noted in the cells growing in the medium with 0.5% and 2% glucose, but in the case of 4% glucose it was higher (Figure 6). This observation may be connected with stimulation of glycolytic flux and fermentation yield. Higher or faster formation of NADH in glycolysis must be reoxidised to NAD^+^, and this transition is catalysed by alcohol dehydrogenases (ADHs). During growth on glucose (fermentative carbon metabolism), Adh1p is almost exclusively responsible for the production of ethanol. Thus, increased NAD^+^ content with increased glucose concentration (Figure 6D) may be the result of higher activity of Adh1p. Indeed, there are studies presenting induction of *ADH1* expression in a glucose-dependent fashion [52] and the correlation between ethanol level and glucose concentration is also well known. The NADPH/NADP^+^ couple connected with the PP pathway and required for assimilatory reactions also shows relevant dependencies. Under fermentative metabolism the level of NADPH is similarly independent of glucose concentration, although the level of NADP^+^ increases with the increased glucose concentration (Figure 6A,C). This may be caused by increased consumption of NADPH due to higher anabolic demands, as indicated by the results of the cellular biosynthetic capabilities obtained in this study (Figure 4 and Figure 5). It has been noted that formation of biomass requires oxidation of NADPH to NADP^+^ in the amount of about 6.5 mmol per 1 g biomass [53]. Moreover, as was shown by Fan et al., most of the NADPH in growing cells is devoted to biosynthesis and the overall demand for NADPH for biosynthesis can even reach 80% of total cytosolic NADPH production [54]. The recently published data also present decreases in the NADPH/NADP^+^ ratio with a simultaneous increase in the NADP^+^ level, which may reflect increased anabolic capabilities [55,56].

Moreover, the highest level of NADPH observed in the medium with glycerol requires clarification (Figure 6A). First of all, NADPH formation involves other metabolic pathways in the presence of glucose and in the glycerol medium. In the presence of glucose, NADPH is generated mainly by the PP pathway. In turn, during growth on glycerol, NADPH is generated by the cytosolic NADP^+^-specific isocitrate dehydrogenase Idp2p or by mitochondrial NADPH-generating systems [13,19]. The high level of NADPH, the low level of NADP^+^ and the low rate of cell population growth may also indicate low biosynthetic capabilities of cells growing on the medium with glycerol (Figure 4, Figure 6A,C). At this point, it is worth emphasizing that the results of the analysis performed on yeast cells cultivated in the medium containing glycerol and low glucose may differ despite the fact that in both cases respiratory metabolism is activated. This is due to the different pathways necessary to utilise glycerol in comparison with glucose. Furthermore, cells growing on glycerol from the beginning have adapted to utilise this carbon source and to the respiratory metabolism implications, whereas cells growing under low glucose conditions gradually change the type of metabolism from respiro-fermentative to fully respiratory. These differences influence gene expression and metabolic pathway activity. As was shown by Mattenberger et al., several transcriptomic changes can be observed in yeast cells during growth in the medium with glycerol. In general, growth in the medium with glycerol up-regulates expression of genes involved in oxidative respiration, ATP synthesis and transmembrane transport. Moreover, several genes connected with cellular biosynthesis, ribosome biogenesis, translation, carbohydrate derivative biosynthesis and protein folding are down-regulated [57]. Our results (Figure 1A, Figure 4A,C, Figure 6A,C,E) are in line with those findings.

It is worth noting that the content of thiol groups in the yeast cells cultivated in the medium with glycerol and glucose is almost at the same level or even higher in the case of mutant strains (Figure 3). The high thiol groups content in the case of respiratory metabolism (Figure 3) may be associated with increase in antioxidant enzymes activity but also with high levels of reducing agents such as NADPH (Figure 6A). NADPH is particularly relevant for proper functioning of the thiol redox system [58] and can be used for reduction of thiol groups. Additionally, the high level of tryptophan (Figure S1), which can be used for de novo NADPH synthesis, may indicate the important role of NADPH in maintaining cellular redox status in the case of respiratory metabolism. Furthermore, NADPH is considered to be a directly operating antioxidant, effective in both scavenging free radicals and repairing biomolecule-derived radicals [18,59], which may also explain the result of lower ROS content noted in the medium with glycerol (Figure 2A).

The relationship between carbon metabolism and redox status is related with energy yield and specific cellular costs and benefits of different types of metabolism, which depends on nutrient availability conditions [22,23,60]. The obtained results confirm the assumption that although fermentation is a less energy efficient metabolic pathway (by taking into account only the amount of obtained ATP), it is more advantageous for faster growth of both individual cells (increase in cell size) and cell population (number of cells), which ensures evolutionary survival of microorganisms. In the medium with glycerol (aerobic respiration), cells generate a high level of ATP, but despite that, the rate of cell growth in the population is lower (Figure 1A, Figure 4 and Figure 5). In turn, for cells conducting fermentative metabolism, a higher rate of cell growth in the population (which depends on cell proliferation) and increase in cell size is observed despite the lower level of ATP (Figure 1A, Figure 4 and Figure 5). Moreover, there is a clear correlation between cell size and glucose concentration in medium (Figure 5). Based on these results, we may assume that the cell size or proliferation effectiveness, or consequently the growth rate of the population, does not depend directly on the ATP level (Figure 1, Figure 4 and Figure 5). However, it should be emphasised that glucose is not only the source of energy; it also provides a carbon skeleton for biosynthesis of many biological molecules. In the cell, glucose utilisation through glycolysis and the PP pathway provides several carbon intermediates required for biosynthesis of essential cellular compounds, inter alia nucleotides, amino acids, lipids and vitamins [29,61,62]. Therefore, in proliferating cells, nutrients seem to be effectively incorporated into the generation of building blocks needed for production of a new cell. When the nutrients are scarce, cells adapt metabolism to obtain the maximum free energy from limited resources and survive in these conditions. In turn, in the presence of abundant resources, they incorporate nutrients into the biomass, which suggests existence of a specific metabolic trade-off [23,62]. An interesting metabolic dependence may be observed in the case of the Δ*sod1* mutant, which exhibits a higher level of ATP in comparison with the WT strain in all analysed conditions (Figure 1A), accompanied by the lower growth of cell population (Figure 4). This suggests that lack of SOD1 may impair the flux between metabolic pathways and thereby the efficiency of the mechanisms responsible for maintaining the cellular homeostasis. This is in line with the reports that yeast strain lacking Sod1p perform an incomplete respiration repression in the presence of glucose [63,64]. Additionally, studies of Reddi and Culotta underline the unconventional signaling role of Sod1p in glucose metabolism. According to these studies, metabolic flux between aerobic respiration and fermentation is partly connected with Sod1p stabilisation of Yck1p and Yck2p (casein kinase 1-gamma (CK1-γ) homologs), which are essential for nutrient sensing. Sod1p binds to the C-terminal domain of these kinases and promotes their stability by catalyzing superoxide conversion to peroxide, which prevents their degradation [63]. Furthermore, the results of Reddi and Culotta and our studies ([7]; Figure 2A), showing high ROS content in glucose conditions where mitochondrial respiration is repressed, provide additional arguments for the existence of non-mitochondrial sources of ROS in the yeast cells.

## 5. Conclusions

To conclude, this study shows that carbon metabolism directly affects the redox status and selected parameters of cellular physiology. These relationships are particularly evident in the case of Δ*sod1* and Δ*sod2* mutants. The lack of Sod1p and Sod2p causes different consequences depending on the conditions varying the mitochondrial activity. This provides an argument for the existence of non-mitochondrial ROS sources in yeast cells. Additionally, the results of the analysis of cellular physiology parameters such as the growth rate of the cell population, cell size, ATP and nucleotide pyridine cofactors content suggest that non-mitochondrial ROS generation can be related to cellular biosynthesis. The presented results and literature data used for their interpretation integrate a wide range of information that might be helpful for understanding of the adaptive capabilities of cells to environmental changes.

## Supplementary Materials

The following are available online at https://www.mdpi.com/2073-4425/11/7/780/s1, Figure S1: The tryptophan content in the WT strain, Δ*sod1* and Δ*sod2* mutants cultivated in respiratory and fermentative condition (medium with 2% glucose). Data are presented as mean ± SD from three independent experiments. ## *p* < 0.01, ### *p* < 0.001 as compared to respiratory metabolism.
